# Risk of Developing Insulin Resistance in Adult Subjects with Phenylketonuria: Machine Learning Model Reveals an Association with Phenylalanine Concentrations in Dried Blood Spots

**DOI:** 10.3390/metabo13060677

**Published:** 2023-05-23

**Authors:** María Jesús Leal-Witt, Eugenia Rojas-Agurto, Manuel Muñoz-González, Felipe Peñaloza, Carolina Arias, Karen Fuenzalida, Daniel Bunout, Verónica Cornejo, Alejandro Acevedo

**Affiliations:** Instituto de Nutrición y Tecnología de Alimentos INTA, Universidad de Chile, Santiago 7830490, Chile

**Keywords:** inborn error of metabolism, glucose metabolism, artificial intelligence, explanatory machine learning

## Abstract

Phenylketonuria (PKU) is an autosomal recessive inborn error of metabolism where high phenylalanine (Phe) concentrations cause irreversible intellectual disability that can be prevented by newborn screening and early treatment. Evidence suggests that PKU subjects not adherent to treatment could be at risk of insulin resistance (IR). We studied how Phe concentrations (PheCs) relate to IR using machine learning (ML) and derived potential biomarkers. In our cross-sectional study, we analyzed subjects with neonatal diagnoses of PKU, grouped as follows: 10 subjects who adhered to treatment (G1); 14 subjects who suspended treatment (G2); and 24 control subjects (G3). We analyzed plasma biochemical variables, as well as profiles of amino acids and acylcarnitines in dried blood spots (DBSs). Higher PheCs and plasma insulin levels were observed in the G2 group compared to the other groups. Additionally, a positive correlation between the PheCs and homeostatic measurement assessments (HOMA-IRs) was found, as well as a negative correlation between the HOMA-Sensitivity (%) and quantitative insulin sensitivity check index (QUICKI) scores. An ML model was then trained to predict abnormal HOMA-IRs using the panel of metabolites measured from DBSs. Notably, ranking the features’ importance placed PheCs as the second most important feature after BMI for predicting abnormal HOMA-IRs. Our results indicate that low adherence to PKU treatment could affect insulin signaling, decrease glucose utilization, and lead to IR.

## 1. Introduction

Phenylketonuria (PKU) is a recessive autosomal disease characterized by the accumulation of phenylalanine (Phe) in the plasma and a decrease in the tyrosine (Tyr) concentration. PKU is mainly caused by mutations in chromosome 12q22-q24.1, which encodes for the enzyme phenylalanine hydroxylase (PAH), which catalyzes the conversion of Phe into Tyr [1]. It is known that high Phe concentrations (PheCs) in the blood are highly neurotoxic, inducing irreversible central nervous system damage [2]. For this reason, PKU should be diagnosed in the neonatal period. International guideline protocol treatment has determined that adequate metabolic control is achieved when a subject maintains a PheC of between 120 and 360 μmol/L [3,4,5]. Conventional treatment is crucial and consists in suspending animal-origin food, such as meat, dairy products and derivatives, fish and seafood, and other foods rich in Phe, such as legumes. Thus, it is necessary to provide patients with a protein substitute without Phe (PS-PheFree)—a mixed amino acid formula—containing enough protein to maintain growth and development within a normal range. In Chile, the newborn screening program (NBS) for PKU began in 1992, presenting an incidence of 1:18,816 NBs with PKU and 1: 10,116 NBs with hyperphenylalaninemia (HPA) [6]. Since then, 280 PKU subjects have been diagnosed, of which 84% were classified as having a classical PKU phenotype. In addition, three frequent variants were identified: c.1162G>A, c.442-?_509+?del, and IVS10-11G>A [7]. When the NBS program started, the diet-therapeutic treatment based on PS-PheFree was subsidized by the government. The subvention covered patients until they reached 18 years of age. However, since 2017, the subvention has been extended for lifelong coverage. Therefore, for 9 years, there were patients that, after reaching 18 years of age, had to start paying for the PS-PheFree treatment, leading to some of them not continuing because of the cost of the treatment and maintaining a vegan diet instead.

The adult PKU population has been increasing over the years, and the scientific evidence highlights that, in adolescence and adulthood, adherence to treatment decreases, affecting life quality [8,9]. At the same time, this poor adherence has been related to neurological consequences [10] and health complications similar to those observed for obesity, hypertension, osteoporosis, and alterations in glucose metabolism [11,12,13]. In 2018, in a multicenter cross-sectional study of 83 patients ranging from 4 to 52 years old, Couce et al. analyzed the carbohydrate metabolism in PKU patients who had received a neonatal diagnosis. They reported that 26% of the PKU subjects had altered fasting insulin levels, with HOMA-IR scores that were significantly higher than those of the control group [14]. Until now, no biomarkers for insulin resistance in PKU have been proposed. Moreover, most studies addressing PKU and glucose metabolism employ statistics such as mean comparisons and pairwise correlations, while biomarker investigation often requires sophisticated multivariate or machine-learning-based approaches [15]. Indeed, machine learning (ML) techniques have been helpful in identifying biomarkers for various human diseases [16,17]. In this study, we investigated whether subjects who suspended treatment increased their cardiovascular risk, and whether this may be detected via plasma biomarkers. In this sense, we evaluated how the suspension of conventional treatment affects insulin resistance, and we applied ML techniques to propose new IR biomarkers in the PKU population.

## 2. Materials and Methods

### 2.1. Study Design

A cross-sectional study was conducted from August 2019 to January 2020. The Ethics Committee of the Institute of Nutrition and Food Technology (INTA) of the University of Chile approved the project in July 2019, and it was conducted in accordance with the principles of the Declaration of Helsinki. 

### 2.2. Participant Description

The INTA of the University of Chile serves as the national reference center for the diagnosis, treatment, and follow-up of PKU. For our adult PKU cohort, we invited 24 subjects over 18 years of age to participate in the study, all of whom had received neonatal PKU diagnoses.

We divided the PKU subjects into two groups:

Group 1 (G1): 10 PKU adults who had continued the conventional treatment (Phe-restriction diet and PS-PheFree intake), with follow-up after they were 18 years old;

Group 2 (G2): 14 PKU adults with low adherence to the treatment, considering a poor Phe-restricted diet, who had suspended the PS-PheFree treatment for more than one year;

Group 3 (G3): 24 control subjects without PKU, with similar characteristics to both PKU groups in terms of age, sex, and body mass index (BMI).

All participants signed a written informed consent.

We excluded pregnant women, subjects with another metabolic disease, subjects with late diagnoses of PKU and with moderate or profound intellectual and physical disability, and those who refused to sign an informed consent.

We also excluded two control subjects who showed altered biochemical analyses regarding their glucose metabolism. These individuals were replaced by two other subjects who met the eligibility criteria.

Before the start of the study, each group was characterized with respect to its participants’ adherence to treatment in the two preceding years. To be classified as adherent to treatment, we considered data corresponding to metabolic control (average phenylalanine concentration measured in dried blood spots), attendance at follow-up appointments, and regular intake of protein substitutes without phenylalanine. We observed that G1 had an average of 9 samples with PheCs of 571 ± 227 umol/L. For G2, we observed a corresponding 9 samples with average PheCs of 590 ± 240 umol/L, similar to G1. In relation to the SP-PheFree intake, the median protein intake of this substitute for G1 was 0.84 gr/kg (IQR: 0.36–1.18), covering 66 ± 31% of the total protein intake. In the case of G2, the median protein intake for PS-PheFree was 0.01 gr/kg (IQR: 0–0.51), covering an average of 24 ± 31% of the total protein intake, which was one of the reasons why this group was considered nonadherent to the treatment (their low PS-PheFree intake or its definitive suspension).

### 2.3. Anthropometric Assessment

We measured subjects’ weight (kg) and height (m) with a Seca scale (0.005 kg accuracy) and stadiometer (0.01 cm margin of error), respectively. Waist circumference (WC) was measured at the middle point between the last rib and iliac crest and expressed in cm. Body mass index (BMI) (kg/m^2^) was also calculated.

### 2.4. Biochemical Analysis

An amount of 15 mL of blood was collected from each patient after an 8–12-hour fast, following the protocol indicated by the external certified laboratory where the analysis of samples was performed. The parameters evaluated were the following: complete blood count, glycemia, insulin, and lipid profile. In the case of insulin and glycemia, three fasting samples were collected per patient at 15-minute intervals. The average value for each variable was then calculated and utilized in the analysis.

#### Homeostasis Model Assessment (HOMA)

Each patient’s HOMA was calculated using the software provided by the University of Oxford (accessed on 1 March 2020; https://www.dtu.ox.ac.uk/homacalculator/), which considered the complete formula published by Matthews [18]. This method assesses β-cell function (HOMA-β%) and insulin sensitivity (HOMA-S%). The reference value used for the altered HOMA-IR was a cut-off of 2.6 [19,20]. Insulin resistance was also assessed using the quantitative insulin sensitivity check index (QUICKI) (1/log10 basal insulin (uIU/mL) + log10 basal glucose (mg/dL)) [21].

### 2.5. Amino Acid and Acylcarnitine Determination by Tandem Mass Spectrometry (MSMS)

The MSMS analysis of amino acids and acylcarnitines was measured in a single 3.2 mm punch disc obtained from individual dried blood spots (DBSs). A single disc was punched out from the DBS into a microplate well containing 100 μL of a working solution (isotopically labelled metabolites in methanol 80%, oxalic acid 0.5 M, and hydrazine 0.06% *v*/*v*). The plate was shaken in an incubator/shaker (Wallac NCS incubator, Perkin-Elmer; Wallac Oy, PO Box 10, 20101 Turku, Finland) at 45 °C for 45 min at a speed of 750 rpm. Then, 75 μL of the well content was transferred to another microplate. Analytical measurements were performed in the multiple reaction monitoring mode (MRM). In order to monitor the performance of our assays, quality control (QC from the Centers for Disease Control and Prevention (CDC)) was performed on the samples in the same plate. The mass spectrometric detection of each analyte was performed using a Micromass Quattro Micro triple-quadrupole mass spectrometer (Waters Corporation, Milford, MA, USA) operating in the positive electrospray ionization mode. The capillary voltage was 3.3 kV. A 40 V cone voltage was used for all analytes. The source temperature was 120 °C and the desolvation temperature was 400 °C. Each sample was injected by an autosampler (Waters 2777 C, Waters Corporations, Manchester, UK) and eluted by an HPLC pump (Waters 1525 µ) at a flow rate of 0.07 mL/min for 2 min. For quantification, the instrument was operated in MRM mode at the unit resolution. A 50 ms dwell time was used between the transitions. A 10 eV collision energy was used for the collision-induced dissociation.

### 2.6. Statistical Analysis

The distribution of variables was assessed using the Shapiro–Wilk test. Normally distributed variables are presented as means with 95% confidence intervals (CIs), while non-normally distributed variables are reported as medians with interquartile ranges (IQRs). Differences among G1, G2, and G3 patients for normally distributed variables were evaluated using ANOVA tests, followed by Student’s *t*-tests for group differentiation. Non-normally distributed variables were examined using the Kruskal–Wallis test, followed by the Mann–Whitney U test for group differentiation. As most variables exhibited a non-normal distribution and considering the number of patients, a nonparametric analysis was assumed. Spearman’s correlations were conducted to measure the dependence between numerical variables. A *p*-value of 0.05 was considered significant. Statistical analysis was carried out using JMP 16.2.0.

### 2.7. Machine Learning Model

We implemented a k-fold cross-validation approach to partition our dataset into training and test sets. A 3-fold grouping was used to ensure sufficient training samples per cohort, while intentionally avoiding oversampling to prevent model overfitting. Importantly, we anticipated the presence of “leaker” features directly related to our target variable, such as the HOMA-IR, fasting insulin, and QUICKI, and consequently masked these variables in our prediction model. Of note, a “leaker” feature in machine learning refers to data that unintentionally include information from the target variable, therefore providing a misleadingly high performance during training and validation but leading to poor results in the test set. We selected the XGBoost algorithm [22] to account for the small sample size and address class imbalance issues. This algorithm combines bagging and boosting techniques to generate a model, involving bagging a random subset of training data and its features for multiple instances of the model, averaging the predictions, and using a sequence of weak learners to correct the errors of the previous learner iteration. To avoid assigning all the model weight to a single feature, we performed Ridge regularization (L2), conducting a hyperparameter search across several XGBoost parameters, including the alpha (L1 regularization), lambda (L2 regularization), learning rate, maximum depth (lowered to avoid overfitting), and scale_pos_weight (accounting for unbalanced positive samples). We constructed a range of 10k models from the hyperparameter sampling and selected those with an AUC accuracy above 0.9 to extract the most informative features. To obtain an agnostic weight assigned to the features, we used SHAP values, based on the concept of Shapley values [23]. Finally, we ranked the informative features based on their computed weights. Our code was implemented in Python (https://www.python.org/ accessed on 1 March 2020), plots were generated using Plotly (https://plotly.com/ accessed on 1 March 2020), and hyperparameter fine-tuning, sampling, and the detection of the Pareto front using the NSGAIISampler were performed using Optuna (https://optuna.org/ accessed on 1 March 2020).

### 2.8. Code Availability

All results pertaining to machine learning can be reproduced using the code available at https://github.com/DeepenData/Phenylketonuria_IR_and_Machine_Learning accessed on 1 March 2020.

## 3. Results

A total of 24 adult patients with PKU were included in this analysis, and 42% of the patients were female, ranging from 18 to 30 years old. All of them were diagnosed in the neonatal period, and according to the diagnostic Phe, they were classified as classical PKU. Fourteen (58%) had suspended the PS-PheFree treatment and discontinued follow-ups (the G2 group). All PKU adults were compared with the control group (the G3 group), without differences in age, sex, and BMI (Table 1).

Anthropometrically, the groups were compared in relation to the BMI, obtaining an average for the complete group of 26.6 ± 5.4 kg/m^2^ (95% CI: 25.1–28.2 kg/m^2^), with no differences between the groups. Nonetheless, G2 presented higher BMIs and waist circumferences (Table 1).

In relation to the biochemical analysis, a significant difference in glycemia levels was observed between G1 and G3 (Table 2). Concerning plasmatic insulin, G2 presented an average concentration of 17.8 ± 13.2 µIU/mL, which was over the reference value indicated by the certificated laboratory (≤16 µIU/mL), and a significant difference compared to G1 (10.2 ± 7.8 µIU/mL) was observed (Table 2). Regarding the lipid profile, we did not observe a significant difference between the groups (Table 2).

To evaluate the IR presence, we observed that the HOMA-IR and HOMA-β scores for G2 presented higher proportions, with a significant difference compared to G1 and G3 in the first index, and a significant difference with only G3 for the HOMA-β scores (*p*-value of <0.05) (Table 2). After the variable distribution analysis, we observed a non-normal distribution for HOMA-IRs represented in the data for the median and IQR values (Table 2). Despite this, we observed that the average (±SD) values of this variable had evidently higher values in G2 compared to the others. The following median values were obtained for the HOMA-IRs from each group: G1: 1.3 ± 0.9 (CI 95%: 0.6–2.0); G2: 2.2 ± 1.6 (CI 95%: 1.3–3.1); and G3: 1.6 ± 0.9 (CI 95%: 1.2–2.0).

For the insulin secretion sensitivity (HOMA-S%), the best percentage was observed in G1, with significant differences compared to G2 and G3 (*p*-value of <0.05), and for the analysis of the QUICKI index, there was a significant difference between G1 and G2 (Table 2).

Thirty-three metabolites were analyzed in the amino acid and acylcarnitine profiles, and we observed significant differences for four amino acids and four acylcarnitines (Table S1). The PheCs were significantly different among the groups (*p*-value of <0.05), with an important difference between G1 (406.4 ± 306.9 umol/L, CI 95%: 186.9–625.9) and G2 (771.0 ± 306.2 umol/L, CI 95%: 594.3–947.8). There was no difference found for the tyrosine concentrations, even in comparison with G3. Regarding the other amino acids, we observed that the leucine concentrations were higher in G3 (110.25 ± 20.69 umol/L, CI 95%: 101.93–118.57) and the glycine and methionine concentrations were higher in G2 (258.47 ± 89.19 umol/L, CI 95%: 206.97–309.97 and 26.17 ± 12.72 umol/L, CI 95%: 18.81–33.51, respectively) (Table S1). For the acylcarnitine concentrations, we primarily found long-chain acylcarnitine, and G3 presented the largest concentrations.

**Figure 1:**
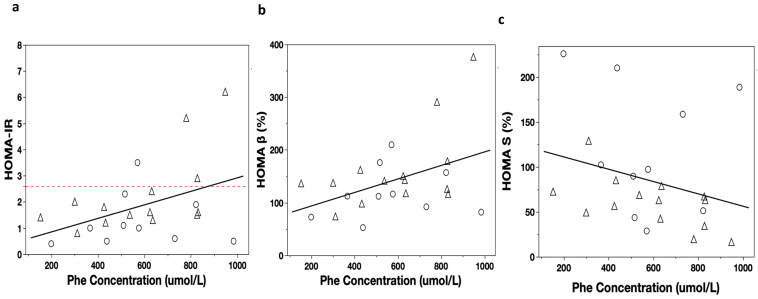
HOMA index correlation with Phe concentrations (PheCs) (umol/L) and Spearman’s correlation: (**a**) PheCs vs. HOMA-IR: *p*-value of 0.02, r = 0.47, CI 95%: 0.08, 0.73; (**b**) PheCs vs. HOMA-β: *p*-value of 0.037, r = 0.43, CI 95%: 0.03, 0.71; (**c**) PheCs vs. HOMA-S%: *p*-value of 0.02, r = −0.47, CI 95%: −0.73, −0.08. Significant *p*-value of <0.05; G1: PKU subjects who continued treatment and PS-PheFree intake, represented by circles; G2: PKU subjects with low adherence, represented by triangles; HOMA-IR: homeostatic model assessment for insulin resistance; HOMA-β: homeostatic model assessment for pancreatic β-cell function; HOMA-S: homeostatic model assessment for sensitivity to insulin secretion.

Positive correlations were observed between the PheCs and fasting plasmatic insulin levels (*p*-value of 0.01; r = 0.51; CI 95%: 0.07–0.73), HOMA-IR index (*p*-value of 0.01; r = 0.50; CI 95%: 0.08–0.73), and HOMA-β index (*p*-value of 0.02; r = 0.48; CI 95%: 0.03–0.71) (Figure 1a,b). Negative correlations were found between the PheCs and HOMA-S% index (*p*-value of 0.01; r = −0.51; CI 95%: −0.73, −0.08) (Figure 1c) and QUICKI (*p*-value of 0.01; r = −0.50; CI 95%: −0.75, −0.11).

**Figure 2:**
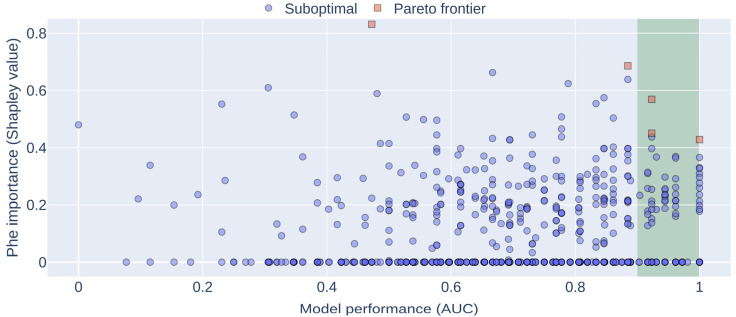
Relationship between phenylalanine importance and model performance. Multiple model instantiations were executed to explore the relationship between the importance of the Phe concentrations (PheCs) and model performance. The importance of PheCs corresponds to its Shapley value concerning the occurrence of abnormal HOMA-IRs, while performance is the area under the curve (AUC) of the receiver operating characteristic (ROC) curve computed on testing data. Squares: Models belonging to the Pareto front. These models have values that other models cannot improve. Circles: Suboptimal models. These have values that other models can improve. Shaded rectangle: High-performing models with AUCs greater than 0.9.

Up to this point in the study, we have shown an association between blood levels of Phe and insulin resistance (IR). Subsequently, we considered a panel of metabolites, including amino acids and acylcarnitines, to investigate their potential as biomarkers of IR in PKU using a machine learning (ML) approach. Subjects with HOMA-IR values exceeding the cut-off of 2.6 were categorized as abnormal [19,20], and an ML model was trained to detect them. We then determined the extent to which each metabolite (feature) from the panel contributes to detecting abnormal HOMA-IRs, with high-contributing features representing potential biomarkers of IR in PKU. Notably, because we had already established an association between Phe and IR, we expected this analysis to confirm such a relationship in terms of Phe’s high contribution to identifying patients with IR. Importantly, small datasets such as ours may lead to variance in the model performance, necessitating adequate regularization and an exploration of the attainable performance range. In this regard, we generated multiple models, with each model trained using cross-validation, bagging, and internal regularization, while computing the AUC on testing data (details in the Section 2). Figure 2 shows the importance of Phe concentrations (PheCs) for predicting abnormal HOMA-IRs as a function of the model performance (measured as AUC), with each circle or square representing a model. As observed in Figure 2, despite the variance in the model performance, the high contribution of the PheCs was predominantly consistent across different models, with a tendency to decrease at high AUCs. These results confirm the association between Phe levels and IR. Furthermore, we identified the models comprising the Pareto front between the Phe importance and AUC (red squares in Figure 2; details in the Section 2), thus ensuring coverage of the entire model variance. We then defined a set of high-performing models, consisting of those with an AUC greater than 0.9 (shaded rectangle in Figure 2). This set of models encodes a robust generalization of the characteristic patterns of IR and was used for further feature importance analysis.

**Figure 3:**
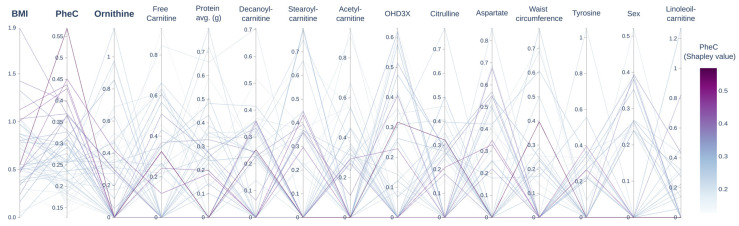
Consensus ranking of feature importance for predicting abnormal HOMA-IRs. This parallel-coordinates plot illustrates the consensus ranking of the feature importance for predicting abnormal HOMA-IRs, with each axis representing a given feature (metabolite) scaled to the range in which this was used. The consensus feature importance was determined by averaging feature rankings across all models with an AUC (area under curve) greater than 0.9. The BMI (body mass index) is identified as the most important feature for predicting an abnormal HOMA-IR, followed by the concentration of phenylalanine. Additionally, several amino acids and acylcarnitines significantly contribute to predicting an abnormal HOMA-IR. We display only the first 15 features, as less-informative features are not used. It should be noted that there are several “paths” that a model can utilize to achieve correct classification, and these paths may focus on different features for doing so. Therefore, consensus ranking represents the most frequently used feature for classification. PheC: concentration of phenylalanine; protein avg. (g): grams per day of total protein.

Subsequently, we obtained the ranking of the feature importance for each high-performing model. Figure 3 displays all the obtained rankings; each line corresponds to a model, and features are sorted by the average importance across models. On average, BMI is the most important feature for predicting an abnormal HOMA-IR score, with PheCs in second place (Figure 3). The predominance of BMI as the top-ranking feature indicates that predictions heavily rely on this variable to determine whether or not a patient’s state will be labeled as an abnormal HOMA-IR and prioritize using Phe as the second reliable variable to combine with the BMI. Overall, these results suggest that the BMI and PheCs are crucial for identifying patients with IR. Additionally, ornithine was in third place, being a strong candidate for an IR biomarker in PKU, along with free carnitine, which was in fourth place (the complete ranking of the feature importance can be found in Figure S1).

## 4. Discussion

Phenylketonuria is one of the most prevalent inborn errors of metabolism; however, the prevalence is not exceedingly high. Worldwide, it is 1:10,000 newborns, and in Chile, the PKU prevalence is 1:18,816 newborns [6]. Due to its relatively low prevalence, it is challenging to include more patients in this type of study. In this analysis, we considered 30% of the adult cohort from this period. In several PKU cohorts, it has been observed that as age increases, adherence to treatment and follow-up lowers [8,9,24,25], and for this reason, the evidence suggests implementing the transition starting at 12 years of age, mainly to prepare subjects to transition from the pediatric stage to the adult stage [3,26]. Further, with the size of the adult PKU population increasing, more cardiometabolic disorders have appeared, which is similar to the general population [27,28]. One of the risk factors involved for the general population is the presence of obesity, which also affects PKU subjects. In the last Chilean PKU cohort update, 43% of subjects were overweight or obese, and the adult group had the major prevalence (43% over 18 years old) [9]. Rodrigues et al. [29], in their systematic review and meta-analysis, observed that there was no relationship between Phe dietary restriction in PKU subjects and BMI; this association appeared when considering only classical PKU subjects, and especially when the PheCs were elevated [29]. When evaluating these 24 PKU subjects, both those who were adherent and those who were not adherent to treatment, we observed in 54% an excess of weight (23% were obese). For reasons such as this, the guidelines suggest that PKU patients should undergo periodic follow-up with a multidisciplinary team to prevent the appearance of other risk factors [5,29]. Burton et al. [12] analyzed the comorbidity prevalence in PKU subjects, observing a high prevalence of renal failure (with or without hypertension), being overweight, and other conditions, and to a lesser degree, diseases such as osteoporosis [12]. Furthermore, Trefz et al. [13] evaluated the presence of comorbidities in a German adult cohort with PKU, considering subjects with early and late PKU diagnoses. Here, when they separated the groups considering the time of diagnosis, they observed predominant conditions in early PKU that preceded the comorbidities described in late PKU. For this reason, they postulated that the elevated PheCs caused by nonadherence or abandonment of treatment, which were similar to the concentrations observed in the late PKU subjects, were triggering the emergence of signs, symptoms, and comorbidities that may have stimulated the development of cardiovascular disease [13]. When analyzing the biochemical statuses of the PKU subjects, we did not observe a significant difference between the groups concerning the lipid profile. However, we observed an alteration in the glucose metabolism. Without an alteration in fasting plasmatic glucose, the plasmatic insulin was higher in the group that had suspended conventional treatment. As such, we calculated the updated HOMA indices provided by Oxford University (https://www.dtu.ox.ac.uk/homacalculator/accessed on 1 March 2020) in 2002, complementing the HOMA-IR score with the function of pancreatic β-cells for insulin secretion (HOMA-β%) and the sensitivity to the secreted insulin (HOMA-S%). This update accounts for variations in hepatic and peripheral resistance. While the HOMA-IR is commonly used, we also calculated the other indices and complemented the results by calculating the QUICKI index, observing the same tendencies. The most affected group was G2, and these results suggested that, to maintain glucose stability, the functionality of the pancreatic β-cells in these subjects was increased, but their sensitivity to secreted insulin was low. In 1980, Stewart et al. described that chronic hyperphenylalaninemia could indicate a pancreas subsensitivity in response to insulin after a high load of Phe. This study was carried out on PKU subjects with late diagnoses who had irreversible intellectual disability caused by high PheC exposure. Their conclusion was as follows: “After an acute high load of Phe in the PKU patients exists an absence of an increased secretion of insulin which suggests that a chronic hyperphenylalaninemia attenuates the insulin response” [30]. In 2015, Kanufre et al. [31] observed a positive correlation between plasmatic insulin and the HOMA-IR index in overweight PKU patients ranging from 4 to 15 years old. In 2018, Couce et al., in a multicenter study, analyzed the glucose metabolism of 83 PKU subjects, and they reported plasmatic insulin levels and HOMA-IR scores that were greater than those of the control group in 26% of the subjects [14].

It is known that the secreted insulin by pancreatic β-cells is regulated by glucose concentrations, encouraging nutrient absorption. The insulin signaling cascade results from the binding of this enzyme to its IRα receptor, which activates the tyrosine kinase activity of the receptor β subunit (IRβ), causing the autophosphorylation of this tyrosine domain kinase and triggering the signaling cascade to promote glucose uptake via GLUT-4 [32]. It has been described that any failure in any of these cascade steps can lead to a defect in glucose uptake, favoring the development of diabetes mellitus 2 (T2D) [32]. It is known that some metabolites, such as fatty acids, can alter this signaling pathway at different stages of the cascade. Likewise, some branched and aromatic amino acids are associated with IR, and high concentrations have been observed in subjects with T2D. However, it is not yet clear how they are involved. Zhou et al. [33] recently suggested the role that the PheC could play, trying to elucidate whether its increase is a cause or consequence of T2D. In the first stage of their work, an animal model (C57BL/6j wild mice) was given a feed chow with a higher Phe content for 12 weeks, increasing the amount of PheCs to 140 μmol/L. There was no difference in caloric intake, but the following consequences were observed: increased fasting glucose levels, impaired glucose tolerance tests, higher insulin concentrations, altered HOMA-IR scores, and decreased tolerances to insulin. These consequences, which resulted after 6 months on this diet, led to the development of IR and T2D. In the second stage, a cell culture model (murine adipocytes and rat skeletal myoblasts) was supplemented with methyl-phenylalanine (Met-Phe), increasing six-fold the intracellular Phe content. It was observed that Met-Phe prevented the insulin-stimulated glucose uptake, suggesting that the PheC attenuates insulin signaling intracellularly, confirming its role in decreasing tyrosine domain phosphorylation in the IR receptor (the receptor of the insulin 1 (IRS1) substrate, among other insulin signaling indicators). In addition, it was observed that Met-Phe supplementation prevented the enrichment of GLUT-4 in the adipocyte membrane [33]. Given these results, we therefore considered that the high concentrations of Phe in the adult subjects who had not adhered to treatment could not only cause neurological and behavioral alterations in this age group, but could also affect the insulin signaling pathway, altering glucose metabolism. These results and the evidence found in the literature led us to hypothesize that subjects with PKU could be more susceptible to the development of IR due to their condition. Through machine learning, several models were created and tasked to identify subjects with altered HOMA-IR scores, considering the established cut-off point of 2.6 [19,20], and to analyze how other variables contributed to this classification [20,34,35]. We selected those models that presented an AUC greater than 0.9, which were considered the most accurate. From these, we determined that the variables that contributed more to identifying a subject with altered HOMA-IR scores were the BMI (first) and PheC (second) The positive association between BMI and the development of insulin resistance has been very well described in the literature, supporting our approach [36,37]. Then, focusing on the PheCs, we observed that despite the high contribution of the BMI, they appeared to be a heavy contributor. In the third and fourth positions of importance were ornithine and free carnitine, respectively. Ornithine may serve as a biomarker for IR in PKU, as it has been described that it can activate membrane depolarization in pancreatic islet β-cells, leading to increased cytosolic Ca^2+^ levels and the subsequent stimulation of insulin secretion [38]. Thus, higher levels of ornithine may compensate for insulin resistance. Moreover, abnormal carnitine regulation has been associated with mitochondrial dysfunction and IR [39], and carnitine supplementation has been proposed as a strategy for managing insulin resistance and type 2 diabetes [40]. Consequently, carnitine may also be considered as a potential biomarker. The application of machine learning in investigating PKU and IR has not been addressed in previous studies, making this work a pioneer in analyzing this association by considering not only the Phe levels but also a set of metabolic and anthropometric variables, and confirming the importance of Phe over other amino acids. These results open a new line of research that will allow us to determine whether the PheC has a deleterious effect on glucose metabolism, and whether this influence in the development of IR could finally lead to type 2 diabetes.

## 5. Conclusions

Machine learning models are primarily employed to predict the occurrence of diseases or conditions based on measurements, such as metabolite abundance or biochemical tests. However, along with prediction, machine learning models can provide explanations in terms of feature importance for making predictions, which helps gain insight into how features relate to the target variable, which, in our case, was the abnormal HOMA-IR. We showed that employing an explanatory-based strategy can reveal latent issues underlying the condition. Specifically, low adherence to treatment in PKU subjects, allowing for high Phe concentrations maintained over time, can not only cause neurological alterations in executive function and behavior, as already described in the literature, but can also affect insulin regulation, decreasing glucose utilization, and facilitating the development of insulin resistance. Machine learning techniques can help PKU treatment, allowing for a multidimensional approach comprising several aspects of the condition, such as those already mentioned. Moreover, our approach can improve the understanding of the complex relationships between measurements and health outcomes. As a result, it may facilitate better decision making by clinicians and researchers.

## Figures and Tables

**Table 1:** Demographic and anthropometric characterization.

	G1 Group (n = 10)	G2 Group (n = 14)	G3 Group (n = 24)	*p*-Value
Age (years)	24 (19–27)	24 (18–26)	23 (19–26)	NS **
Sex (F/M)	5:5	5:9	11:13	NS ***
Weight (kg) ^b^	65 (58–94)	75 (59–81)	68 (61–83)	NS **
Height (m) ^a^	163 ± 9 (157–170)	163 ± 8 (158–168)	165 ± 9 (161–168)	NS *
BMI (kg/m^2^) ^a^	26 ± 5 (23–30)	28 ± 7 (24–32)	26 ± 5 (24–28)	NS *
Waist circumference (cm) ^b^	82 (76–97)	92 (78–103)	81 (75–91)	NS **

G1: PKU subjects who continued treatment and PS-PheFree intake; G2: PKU subjects who suspended treatment and PS-PheFree intake; G3: control group; BMI: body mass index; NS: not significant. ^a^ normal distribution: values represented by the median ± SD and a 95% confidence interval (CI); ^b^ non-normal distribution: values represented by the median and an interquartile range (IQR: Q1–Q3); * ANOVA test; ** Kruskal–Wallis test; *** Fisher’s test; n = number of subjects.

**Table 2:** Biochemical analysis.

	G1 Group (n = 10)	G2 Group (n = 14)	G3 Group (n = 24)	*p*-Value
Glycemia (mg/dL) ^a^	85.7 ± 5.5 (81.8–89.7)	90.7 ± 4.9 (87.8–93.4)	92.2 ± 8.5 (88.6–95.8)	<0.05 ^ɛ^
Insulin (µIU/mL) ^b^	7.9 (4.1–15.9)	12.4 (10.6–19.4)	9.6 (7.1–14.7)	<0.05 ^ɣ^
HOMA-IR ^b^	1.0 (0.5–2.0)	1.6 (1.4–2.5)	1.3 (0.9–1.9)	<0.05 ^ɣǂ^
HOMA-β (%) ^b^	112.2 (79.7 –61.7)	139.1 (117.0–166.0)	107.5 (90.3–127.5)	<0.05 ^ǂ^
HOMA-S (%) ^a^	119.5 ± 71.6 (68.3–170.7)	60 ± 28.6 (43.6–76.7)	82.8 ± 37.5 (66.9–98.7)	<0.05 ^ɣɛ^
QUICKI ^a^	0.36 ± 0.04 (0.33–0.39)	0.32 ± 0.02 (0.31–0.33)	0.34 ± 0.03 (0.33–0.35)	<0.05 ^ɣ^
Total cholesterol (mg/dL) ^a^	137.1 ± 27.6 (117.3–156.8)	139.6 ± 27.5 (123.7–155.5)	154.1 ± 31.1 (140.9–167.3)	NS *
HDL cholesterol (mg/dL) ^a^	50.8 ± 12.8 (41.5–59.9)	44.3 ± 8.8 (39.2–49.4)	49.9 ± 9.3 (45.9–53.9)	NS *
LDL cholesterol (mg/dL) ^b^	65.6 (48.7–82.4)	68.3 (62.2–83.8)	77 (60.2–99.4)	NS **
Triglycerides (mg/dL) ^b^	81.5 (54–115)	93.5 (60–164)	92.5 (79–122)	NS **

G1: PKU subjects who continued treatment and PS-PheFree intake; G2: PKU subjects who suspended treatment and PS-PheFree intake; G3: control group; BMI: body mass index; NS: not significant; ^a^ normal distribution: values represented by the median and a 95% confidence interval (CI); ^b^ non-normal distribution: values represented by the median and an interquartile range (Q1–Q3); * ANOVA test; ** Kruskal–Wallis test; ^ɣ^ G1 and G2 significant difference; ^ɛ^ G1 and G3 significant difference; ^ǂ^ G2 and G3 significant difference; n = number of subjects.

## Data Availability

The data can be obtained by contacting the first author, M.J.L.-W. Data is not publicly available due to privacy.

## Supplementary Materials

The following supporting information can be downloaded at https://www.mdpi.com/article/10.3390/metabo13060677/s1, Table S1: Acylcarnitine and Amino Acid Profile comparison. Figure S1. Complete ranking of feature importance.

## References

[1] Marta Colombo C., Verónica Cornejo E., Erna Raimann B. (2017). Errores Innatos en el Metabolismo del Niño.

[2] Vockley J., Andersson H.C., Antshel K.M., Braverman N.E., Burton B.K., Frazier D.M., Mitchell J., Smith W.E., Thompson B.H., The American College of Medical Genetics and Genomics Therapeutic Committee (2014). Phenylalanine hydroxylase deficiency: Diagnosis and management guideline. Genet. Med..

[3] Singh R.H., Cunningham A.C., Mofidi S., Douglas T.D., Frazier D.M., Hook D.G., Jeffers L., McCune H., Moseley K.D., Ogata B. (2016). Updated, web-based nutrition management guideline for PKU: An evidence and consensus based approach. Mol. Genet. Metab..

[4] Castro G., Hamilton V., Cornejo V. (2017). Chilean Nutrition Management Protocol for Patients With Phenylketonuria. J. Inborn Errors Metab. Screen..

[5] MacDonald A., van Wegberg A.M.J., Ahring K., Beblo S., Bélanger-Quintana A., Burlina A., Campistol J., Coşkun T., Feillet F., Giżewska M. (2020). PKU dietary handbook to accompany PKU guidelines. Orphanet J. Rare Dis..

[6] Cornejo V., Raimann E., Cabello J.F., Valiente A., Becerra C., Opazo M., Colombo M. (2010). Past, present and future of newborn screening in Chile. J. Inherit. Metab. Dis..

[7] Hamilton V., Santa María L., Fuenzalida K., Morales P., Desviat L.R., Ugarte M., Pérez B., Cabello J.F., Cornejo V. (2017). Characterization of Phenyalanine Hydroxylase Gene Mutations in Chilean PKU Patients. JIMD Rep..

[8] Burton B.K., Leviton L. (2010). Reaching out to the lost generation of adults with early-treated phenylketonuria (PKU). Mol. Genet. Metab..

[9] Leal-Witt M.J., Salazar M.F., Peñaloza F., Castro G., Hamilton V., Arias C., Peredo P., Valiente A., De la Parra A., Cabello J.F. (2021). Update on Dietary Compliance, Nutritional Status, and Neuropsychological Functioning in the Chilean Phenylketonuria cohort. J. Inborn Errors Metab. Screen..

[10] Burlina A.P., Lachmann R.H., Manara R., Cazzorla C., Celato A., van Spronsen F.J., Burlina A. (2019). The neurological and psychological phenotype of adult patients with early-treated phenylketonuria: A systematic review. J. Inherit. Metab. Dis..

[11] Rocha J.C., van Rijn M., van Dam E., Ahring K., Bélanger-Quintana A., Dokoupil K., Ozel H.G., Lammardo A.M., Robert M., Heidenborg C. (2016). Weight Management in Phenylketonuria: What Should Be Monitored?. Ann. Nutr. Metab..

[12] Burton B.K., Jones K.B., Cederbaum S., Rohr F., Waisbren S., Irwin D.E., Kim G., Lilienstein J., Alvarez I., Jurecki E. (2018). Prevalence of comorbid conditions among adult patients diagnosed with phenylketonuria. Mol. Genet. Metab..

[13] Trefz K.F., Muntau A.C., Kohlscheen K.M., Altevers J., Jacob C., Braun S., Greiner W., Jha A., Jain M., Alvarez I. (2019). Clinical burden of illness in patients with phenylketonuria (PKU) and associated comorbidities—A retrospective study of German health insurance claims data. Orphanet J. Rare Dis..

[14] Couce M.L., Sánchez-Pintos P., Vitoria I., De Castro M.-J., Aldámiz-Echevarría L., Correcher P., Fernández-Marmiesse A., Roca I., Hermida A., Martínez-Olmos M. (2018). Carbohydrate status in patients with phenylketonuria. Orphanet J. Rare Dis..

[15] Mokou M., Mischak H., Frantzi M. (2023). Statistical determination of cancer biomarkers: Moving forward clinically. Expert Rev. Mol. Diagn..

[16] Basu D., Sinha R., Sahu S., Malla J., Chakravorty N., Ghosal P.S. (2022). Identification of severity and passive measurement of oxidative stress biomarkers for β–thalassemia patients: K-means, random forest, XGBoost, decision tree, neural network based novel framework. Adv. Redox Res..

[17] Sharma A., Verbeke W.J.M.I. (2020). Improving Diagnosis of Depression With XGBOOST Machine Learning Model and a Large Biomarkers Dutch Dataset (n = 11,081). Front. Big Data.

[18] Caumo A., Perseghin G., Brunani A., Luzi L. (2006). New Insights on the Simultaneous Assessment of Insulin Sensitivity and -Cell Function With the HOMA2 Method. Diabetes Care.

[19] Ascaso J.F., Pardo S., Real J.T., Lorente R.I., Priego A., Carmena R. (2003). Diagnosing Insulin Resistance by Simple Quantitative Methods in Subjects with Normal Glucose Metabolism. Diabetes Care.

[20] Burrows R., Correa-Burrows P., Reyes M., Blanco E., Albala C., Gahagan S. (2015). Healthy Chilean Adolescents with HOMA-IR **≥** 2.6 Have Increased Cardiometabolic Risk: Association with Genetic, Biological, and Environmental Factors. J. Diabetes Res..

[21] Katz A., Nambi S.S., Mather K., Baron A.D., Follmann D.A., Sullivan G., Quon M.J. (2000). Quantitative Insulin Sensitivity Check Index: A Simple, Accurate Method for Assessing Insulin Sensitivity In Humans. J. Clin. Endocrinol. Metab..

[22] Chen T., Guestrin C. XGBoost: A Scalable Tree Boosting System. Proceedings of the 22nd ACM SIGKDD International Conference on Knowledge Discovery and Data Mining.

[23] Heese R., Mücke S., Jakobs M., Gerlach T., Piatkowski N. (2023). Shapley Values with Uncertain Value Functions. Proceedings of the Advances in Intelligent Data Analysis XXI: 21st International Symposium on Intelligent Data Analysis, IDA 2023.

[24] Jurecki E.R., Cederbaum S., Kopesky J., Perry K., Rohr F., Sanchez-Valle A., Viau K.S., Sheinin M.Y., Cohen-Pfeffer J.L. (2017). Adherence to clinic recommendations among patients with phenylketonuria in the United States. Mol. Genet. Metab..

[25] Green B., Browne R., Firman S., Hill M., Rahman Y., Kaalund Hansen K., Adam S., Skeath R., Hallam P., Herlihy I. (2019). Nutritional and Metabolic Characteristics of UK Adult Phenylketonuria Patients with Varying Dietary Adherence. Nutrients.

[26] van Spronsen F.J., van Wegberg A.M., Ahring K., Bélanger-Quintana A., Blau N., Bosch A.M., Burlina A., Campistol J., Feillet F., Giżewska M. (2017). Key European guidelines for the diagnosis and management of patients with phenylketonuria. Lancet Diabetes Endocrinol..

[27] Karam P.E., Majdalani M.N., Daher R.T., Barhoumi A., Yazbeck N. (2015). Cardiovascular disease biomarkers in patients with inborn errors of protein metabolism: A pilot study. J. Hum. Nutr. Diet. Off. J. Br. Diet. Assoc..

[28] Azabdaftari A., van der Giet M., Schuchardt M., Hennermann J.B., Plöckinger U., Querfeld U. (2019). The cardiovascular phenotype of adult patients with phenylketonuria. Orphanet J. Rare Dis..

[29] Rodrigues C., Pinto A., Faria A., Teixeira D., van Wegberg A.M.J., Ahring K., Feillet F., Calhau C., MacDonald A., Moreira-Rosário A. (2021). Is the Phenylalanine-Restricted Diet a Risk Factor for Overweight or Obesity in Patients with Phenylketonuria (PKU)? A Systematic Review and Meta-Analysis. Nutrients.

[30] Stewart M.R., Hemli S., Kolodny E.H., Miller A.L., Pallotta J.A. (1980). Carbohydrate Metabolism in Phenylketonuria. Pediatr. Res..

[31] Kanufre V.C., Soares R.D.L., Alves M.R.A., Aguiar M.J.B., Starling A.L.P., Norton R.C. (2015). Metabolic syndrome in children and adolescents with phenylketonuria. J. Pediatr..

[32] Petersen M.C., Shulman G.I. (2018). Mechanisms of Insulin Action and Insulin Resistance. Physiol. Rev..

[33] Zhou Q., Sun W.-W., Chen J.-C., Zhang H.-L., Liu J., Lin Y., Lin P.-C., Wu B.-X., An Y.-P., Huang L. (2022). Phenylalanine impairs insulin signaling and inhibits glucose uptake through modification of IRβ. Nat. Commun..

[34] Muniyappa R., Lee S., Chen H., Quon M.J. (2008). Current approaches for assessing insulin sensitivity and resistance in vivo: Advantages, limitations, and appropriate usage. Am. J. Physiol.-Endocrinol. Metab..

[35] Abdesselam A., Zidoum H., Zadjali F., Hedjam R., Al-Ansari A., Bayoumi R., Al-Yahyaee S., Hassan M., Albarwani S. (2021). Estimate of the HOMA-IR Cut-off Value for Identifying Subjects at Risk of Insulin Resistance Using a Machine Learning Approach. Sultan Qaboos Univ. Med. J..

[36] Friedemann C., Heneghan C., Mahtani K., Thompson M., Perera R., Ward A.M. (2012). Cardiovascular disease risk in healthy children and its association with body mass index: Systematic review and meta-analysis. BMJ.

[37] Chen G., Liu C., Yao J., Jiang Q., Chen N., Huang H., Liang J., Li L., Lin L. (2010). Overweight, obesity, and their associations with insulin resistance and β-cell function among Chinese: A cross-sectional study in China. Metabolism.

[38] Sener A., Best L.C., Yates A.P., Kadiata M.M., Olivares E., Louchami K., Jijakli H., Ladrière L., Malaisse W.J. (2000). Stimulus-secretion coupling of arginine-induced insulin release. Endocrine.

[39] Nowak C., Hetty S., Salihovic S., Castillejo-Lopez C., Ganna A., Cook N.L., Broeckling C.D., Prenni J.E., Shen X., Giedraitis V. (2018). Glucose challenge metabolomics implicates medium-chain acylcarnitines in insulin resistance. Sci. Rep..

[40] Bene J., Hadzsiev K., Melegh B. (2018). Role of carnitine and its derivatives in the development and management of type 2 diabetes. Nutr. Diabetes.

