# Supplementary material for: Risk of Developing Insulin Resistance in Adult Subjects with Phenylketonuria: Machine Learning Model Reveals an Association with Phenylalanine Concentrations in Dried Blood Spots
Source: Metabolites. 2023 May 23;13(6):677. doi: 10.3390/metabo13060677 (PMC10305543; doi:10.3390/metabo13060677)
Supplement: Supplementary file 1 [file metabolites-13-00677-s001.zip › metabolites-2350321-supplementary.pdf]

Supplementary data.

Table S.1 Acylcarnitine and Amino Acid Profile comparison.

	G1 group (n=10)	G2 group (n=14)	G3 group (n=24)	p-value
Alanine (umol/L)[a]	131.04 ± 39.81 (102.56-159.51)	150.1 ± 46.7 (123.1-177.0)	138.2 ± 29.9 (125.6-150.8)	NS *
Aspartate (umol/L)[a]	37.71 ± 9.1 (31.21-44.19)	42.74 ± 15.66 (33.70-51.78)	45.84 ± 16.52 (38.86-52.81)	NS *
Glutamate (umol/L)[b]	34.89 (34.80; 52.49)	47.55 (34.31; 74.41)	52.43 (34.27; 67.79)	NS **
Leucine (umol/L)[b]	78.86 (70.15; 96.31)	84.10 (73.84; 112.81)	109.15 (97.64; 123.56)	<0.05 **[ε ǂ]
Ornitine (umol/L)[a]	38.43 ± 6.66 (33.66-43.19)	33.99 ± 13.28 (26.32-41.65)	42.12 ± 13.28 (36.50-47.71)	NS *
Proline (umol/L)[b]	69.77 (63.11; 87.62)	81.23 (63.90; 98.56)	86.61 (77.74; 113.54)	NS **
Tyrosine (umol/L)[b]	45.68 (35.07; 52.51)	35.98 (32.81; 53.65)	49.23 (41.86; 60.12)	NS **
Free Carnitine (umol/L)[b]	22.20 (18.24; 28.40)	19.67 (17.06; 23.94)	20.44 (16.69; 26.29)	NS **
Propionylcarnitine (umol/L)[a]	1.43 ± 0.71 (0.92; 1.94)	1.40 ± 0.40 (1.16; 1.63)	1.75 ± 0.71 (1.45-2.05)	NS *
Isovalerylcarnitine (umol/L)[b]	0.11 (0.08; 0.12)	0.09 (0.07; 0.11)	0.12 (0.08; 0.14)	NS **
Tiglicarnitine (umol/L)[a]	0.08 ± 0.04	0.05 ± 0.03	0.06 ± 0.04	NS *

	(0.05-0.11)	(0.04-0.07)	(0.05-0.08)	
Me-Glutarylcarnitine (umol/L)[b]	0.06 (0.05; 0.07)	0.06 (0.05; 0.06)	0.06 (0.05; 0.06)	NS **
Decanoylcarnitine (umol/L)[b]	0.10 (0.09; 0.14)	0.11 (0.09; 0.19)	0.16 (0.13; 0.22)	<0.05 **ε
Tetradecanoylcarnitine (umol/L)[b]	0.06 (0.05; 0.07)	0.04 (0.03; 0.05)	0.05 (0.04; 0.06)	NS **
3-OH-Isovalerylcarnitine (umol/L)[b]	0.18 (0.15; 0.21)	0.17 (0.13; 0.23)	0.27 (0.22; 0.29)	<0.05 **ε ɫ
3-OH-Palmitoylcarnitine (umol/L)[b]	0.01 (0.01; 0.02)	0.02 (0.01; 0.02)	0.01 (0.01; 0.02)	NS **
Linoleoylcarnitine (umol/L)[b]	0.88 (0.64; 1.05)	0.63 (0.53; 0.98)	0.87 (0.66; 1.11)	NS **
Arginine (umol/L)[b]	25.61 (20.96; 35.03)	29.66 (21.49; 40.97)	28.02 (23.00; 37.32)	NS **
Citrulline (umol/L)[b]	19.91 (17.66; 23.74)	22.96 (17.50; 26.83)	20.17 (16.62; 25.07)	NS **
Glycine (umol/L)[a]	195.10 ± 45.15 (162.80; 227.40)	258.47 ± 89.19 (206.97; 309.97)	174.36 ± 64.74 (147.02; 201.70)	<0.05 * ɫ
Methionine (umol/L)[a]	19.67 ± 2.97 (17.54; 21.79)	26.17 ± 12.72 (18.82; 33.51)	19.71 ± 3.77 (18.12; 21.31)	<0.05 * ɣ ɫ
Phenylalanine (umol/L)[a]	406.41 ± 306.89 (186.87-625.95)	771.04 ± 306.19 (594.25-947.83)	42.84 ± 6.25 (40.20-45.48)	<0.05 * ɣ ε ɫ
Valine (umol/L)[b]	133.37 (120.70; 165.74)	123.52 (107.12; 152.91)	134.33 (123.36; 161.05)	NS **
Acetylcarnitine (umol/L)[a]	12.97 ± 2.24	12.13 ± 3.30	11.83 ± 3.40	NS *

	(11.37; 14.58)	(10.23; 14.05)	(10.39; 13.27)	
Butyrylcarnitine (umol/L)[b]	0.13<br>(0.10; 0.19)	0.13<br>(0.1; 0.14)	0.12<br>(0.09; 0.17)	NS **
Glutarylcarnitine (umol/L)[a]	0.08 ± 0.03<br>(0.06; 0.10)	0.06 ± 0.03<br>(0.05; 0.08)	0.06 ± 0.03<br>(0.05-0.07)	NS *
Hexanoylcarnitine (umol/L)[b]	0.04<br>(0.04; 0.04)	0.03<br>(0.03; 0.04)	0.04<br>(0.02; 0.04)	NS **
Octanoylcarnitine (umol/L)[b]	0.07<br>(0.06; 0.09)	0.08<br>(0.06; 0.10)	0.09<br>(0.07; 0.10)	NS **
Dodecanoylcarnitine (umol/L)[b]	0.03<br>(0.03; 0.04)	0.04<br>(0.03; 0.06)	0.04<br>(0.03; 0.05)	NS **
Tetradecenoylcarnitine (umol/L)[b]	0.08<br>(0.06; 0.10)	0.08<br>(0.05; 0.13)	0.06<br>(0.05; 0.09)	NS **
Palmitoylcarnitine (umol/L)[b]	0.53<br>(0.46; 0.59)	0.47<br>(0.40; 0.57)	0.63<br>(0.48; 0.79)	NS **
Stearoylcarnitine (umol/L)[b]	0.32<br>(0.27; 0.41)	0.35<br>(0.23; 0.41)	0.42<br>(0.34; 0.49)	<0.05 **[ǂ]
3-OH-Linoleoylcarnitine (umol/L)[b]	0.02<br>(0.02; 0.03)	0.03<br>(0.02; 0.04)	0.04<br>(0.02; 0.05)	<0.05 **[ε]

G1: PKU subjects who continued treatment and PS-PheFree intake; G2: PKU subjects who suspended treatment and PS-PheFree intake; BMI: body mass index; NS: not significant; [a], values represented by the median and a 95% confidence interval (CI); [b], values represented by the median and an interquartile range (Q1 - Q3); *, ANOVA test; **, Kruskal–Wallis test; [¥], G1 and G2 significant difference; [ε], G1 and G3 significant difference; [ǂ], G2 and G3 significant difference.

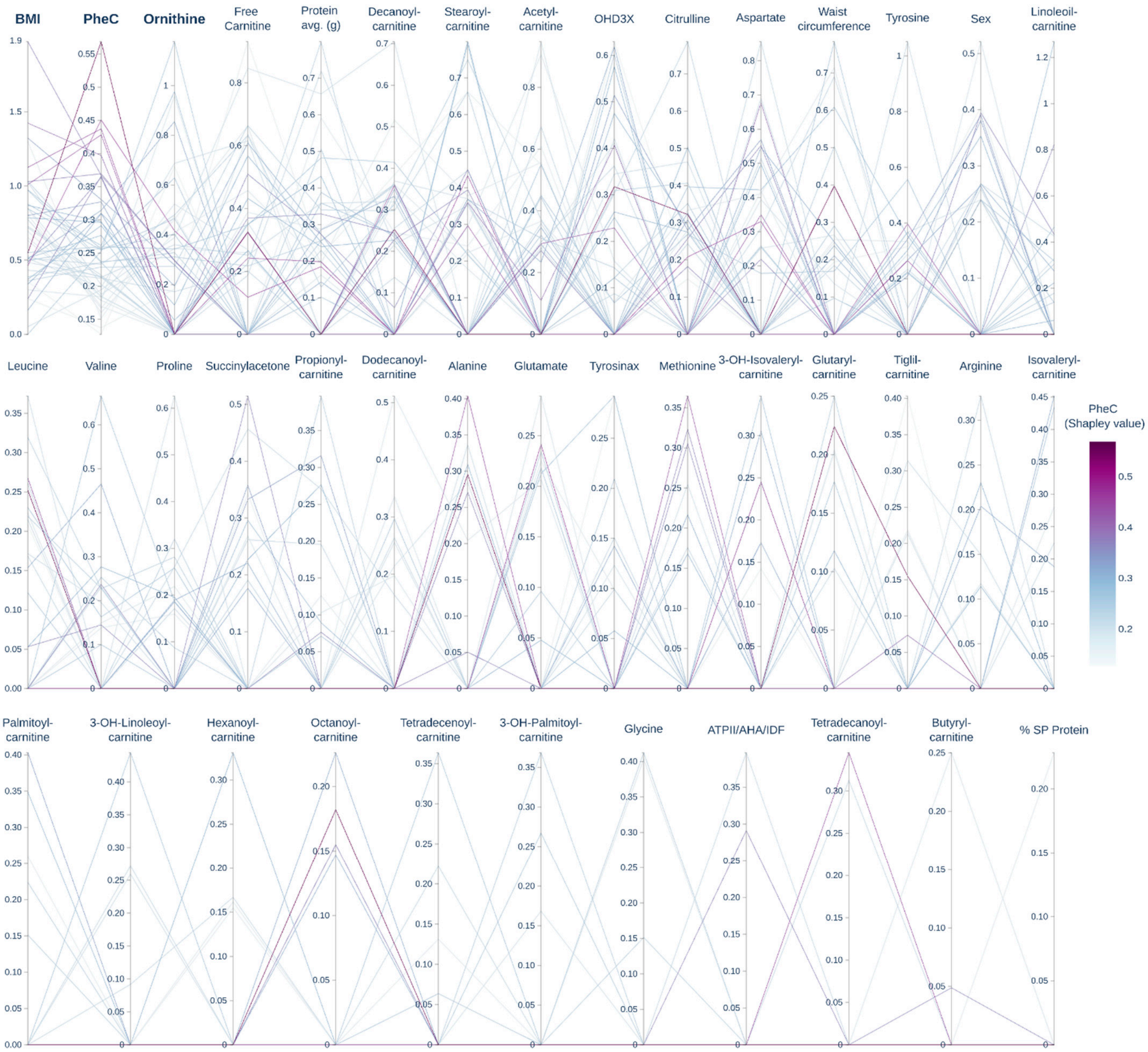

Figure S.1. Complete ranking of features importance for predicting abnormal HOMA-IR. This parallel coordinates plot illustrates the consensus ranking of feature importance for predicting abnormal HOMA-IR, with each axis representing a given feature (metabolite) scaled to the range in which this was used. The consensus feature importance was determined by averaging feature rankings across all models with AUC (Area Under Curve) greater than 0.9. It should be noted that there are several "paths" that a model can utilize to achieve correct classification, and these paths may focus on different features for doing so. Therefore, consensus ranking represents the most frequently used feature for classification.
